# Standardised data collection from people with dementia over the telephone: A qualitative study of the experience of DETERMIND programme researchers in a pandemic

**DOI:** 10.1177/14713012231190585

**Published:** 2023-07-26

**Authors:** Kate Gridley, Josie Dixon, Ben Hicks, Yvonne Birks, Kate Baxter, Eleanor Miles, Louise Robinson, Rotem Perach, Sube Banerjee

**Affiliations:** Social Policy Research Unit, 8748 University of York, UK; Care Policy and Evaluation Centre (CPEC), 4905 London School of Economics and Political Science, London, UK; 12190 Brighton and Sussex Medical School, 1948 University of Sussex, UK; Social Policy and Social Work, 8748 University of York, UK; Social Policy Research Unit, 8748 University of York, UK; School of Psychology, 1948 University of Sussex, UK; Population Health Sciences Institute, Faculty of Medical Sciences, 5994 Newcastle University, UK; School of Social Sciences, 4921 University of Westminster, UK; Faculty of Health, 6633 University of Plymouth, UK

**Keywords:** dementia, data collection, standardised measures, telephone interviews, person-oriented

## Abstract

There is a notable lack of evidence on what constitutes good practice in remote quantitative data collection from research participants with dementia. During the COVID-19 pandemic face-to-face research became problematic, especially where participants were older and more at risk of infection. The DETERMIND-C19 study, a large cohort study of people with dementia, switched to telephone data collection over this period. This paper explores the experiences of researchers who collected quantitative data over the telephone from people with dementia during the first COVID-19 lockdowns in England. The aim was to learn from these experiences, share insights and inform future research practice across disciplines. Seven DETERMIND researchers were interviewed about the processes and challenges of collecting quantitative data from people with dementia over the telephone compared to face-to-face. Data were analysed using reflexive thematic analysis. Two themes were developed: first the telephone adds an extra layer of confusion to an already cognitively complex interaction. Second, researchers found it difficult to recognise subtle cues that signalled participants’ rising emotion over the telephone in time to prevent distress. The researchers employed strategies to support participants which may not have conformed to the strict conventions of structured interviewing, but which were informed by person-oriented principles. Whilst in practice this may be a common approach to balancing the needs of participants and the requirements of quantitative research, it is rare for studies to openly discuss such trade-offs in the literature. Honest, reflective reporting is required if the practice of remote data collection from people with dementia is to progress ethically and with integrity.

## Introduction

Over the last two decades there has been rapid growth in research involving people with dementia (Brooks et al., 2017; Keady et al., 2017; McKeown et al., 2010) and the development of a number of dementia-specific standardised measures to facilitate quantitative data collection, most designed for face-to-face use (Bowling et al., 2015; Cahill et al., 2004; Clarke et al., 2020; Logsdon et al., 2002; Smith et al., 2005).

When the COVID-19 pandemic began in early 2020, research practice in the UK, as across the world, was severely disrupted (Gummer et al., 2020; O’Rouke et al., 2021; Will et al., 2020). The DETERMIND programme (Farina et al., 2020) was in the process of recruiting a cohort of 1000 UK participants newly diagnosed with dementia (and their carers) to complete a wide range of instruments about their attitudes and experiences over three to five years. All quantitative data were originally to be collected face-to-face, but the pandemic prompted a decision to temporarily switch to remote methods. As many health and social services temporarily closed during the pandemic (Wheatley et al., 2022), much routine diagnosis of dementia stopped and baseline recruitment to DETERMIND was also paused. This presented an opportunity for the DETERMIND team to switch their attention to collecting data from participants already consented into the programme to examine the impact of the pandemic and associated lockdowns on quality of life and well-being (DETERMIND-C19).

In addition to qualitative interviews about experiences of the pandemic, quantitative data were collected for DETERMIND-C19 (Hicks et al., 2022; Perach et al., 2023). At this point, telephone interviews had rarely been used to collect standardised data from people with dementia, so alongside DETERMIND-C19 we launched a nested study to enable reflection upon the data collection process. All DETERMIND-C19 researchers collecting quantitative data were invited to take part in a qualitative interview. The purpose of these interviews was to learn about the processes and challenges of collecting standardised quantitative data from people with dementia over the telephone and explore strategies used by researchers to ensure the interviews ran smoothly. In this paper we set out the findings from these qualitative interviews with researchers and explore implications for future studies.

## Literature review

### People with dementia as research participants

Despite the wide recognition of dementia as a global health priority (World Health Organisation, 2017), people with dementia themselves have often been excluded from research participation, including research about dementia, without rationale or discussion (Benson et al., 2020; Silva and Cascio, 2020; Taylor et al., 2012). In England, policy moves have been made to remedy this by encouraging greater involvement in health and social care research (Department of Health, 2015; DH, 2019). A rights based movement has also come to the fore emphasising the moral and ethical imperative for people with dementia to be involved in research that concerns them (Scottish Dementia Working Group Research Sub-Group, UK, 2014; Dementia Action Alliance, 2017). This has been accompanied by a growing literature focussing on ways to foreground the voice of the person with dementia (Swarbrick, 2015; Thoft et al., 2020). However, the methods literature almost exclusively focusses on qualitative methods, emphasising the importance of creativity and adaptation to the varied needs of people with dementia (Cridland et al., 2016; McKeown et al., 2010; Novek & Wilkinson, 2019; Phillipson & Hammond, 2018; Webb et al., 2020). Structured data collection is rarely mentioned in this literature, and where it is this is usually to flag problems with the use of inflexible methods (Keady et al., 2017; Samsi & Manthorpe, 2020).

Nevertheless, people with dementia *are* increasingly and necessarily involved as participants in trials and cohort studies such as DETERMIND. Relatively little has been published about good practice or the experience of participants in this context, with the quantitative methods literature tending to focus on consent and proxy issues (Götzelmann et al., 2021), or the psychometric properties of measures (Bowling et al., 2015; Li et al., 2018; Moniz-Cook et al., 2008; Stoner et al., 2018; Yang et al., 2018). In an opportunistic study of the ‘*unsolicited conversational comments*’ accompanying quantitative data collection, Abendstern et al. (2019) concluded that the interactional variation in real world quantitative data collection should prompt us to ‘*re-examine the complex social context of research encounters more carefully, in effect considering the ‘lived experience’ of completing standardised measures of health and wellbeing*.’ (p. 11). Perfect et al. (2019) interviewed researchers collecting data in care homes and found supportive attitudes, but also concern about the risk of causing distress to some participants who struggled to answer questions or felt they were being ‘tested’. The authors describe a potential conflict between the desire to support the well-being of participants in research and the inflexibility of standardised measure administration.

### The impact of mode on data collection

There is a substantial literature on the influence of interview mode (Fenig et al., 1993; Irvine, 2011; Johnson et al., 2021; Ongena & Dijkstra, 2021). For quantitative data collection in particular, the evidence suggests that, whilst mode of administration can influence data quality (Nandi & Platt, 2017), the largest difference is between questionnaire self-completion (either by hand or online) and administration by an interviewer, regardless of whether this is face-to-face or by telephone (Bowling, 2005; Hope et al., 2014; Ye et al., 2011). It has been shown in studies of cancer patients, for example, that psychological aspects of quality of life may be scored more positively when measures are administered in an interview than when they are self-completed (Cheung et al., 2006).

Some standardised measures have been validated for use with older people over the telephone, but those with cognitive impairment are often excluded from such studies and the experiences of participants tend not to feature at all (McPhail et al., 2009; Senior et al., 2007). We know that survey interviews with older respondents can be more complex interactions than those with younger people, and this may be linked to age related cognitive changes (Beullens et al., 2019), but specific evidence about older people with dementia responding to surveys in different ways is sparse. In 2002, Mason and Wilkinson described piloting telephone interviews with people with dementia for quantitative data collection, but later abandoned this in favour of an approach more ‘*flexible to the needs of the respondents*’ (p. 188). Nearly two decades on, Lawrence et al. (2022) note that remote *qualitative* methods involving older people with mental ill health are still in their infancy, but make no mention of remote *quantitative* methods.

During the COVID-19 pandemic, when face-to-face data collection became impossible, some ongoing dementia studies chose not to collect data from participants with dementia at all (Daley et al., 2022), whilst others shifted to qualitative methods (O’Rourke et al., 2021). For DETERMIND-C19 we opted to attempt remote quantitative data collection with people with dementia, and to monitor and reflect upon this process. The aim was to collate and disseminate insights so that others could learn from and build upon our experiences.

## Methods

### DETERMIND-C19 data collection

From July to October 2020, researchers in three sites across England re-contacted a purposively sampled sub-group of DETERMIND participants inviting them to take part in an additional quantitative interview for DETERMIND-C19. Participants with dementia all had capacity to give informed consent and were given the choice of a telephone or video-call. The measures used were the same combination of dementia specific and more general validated measures used for the full DETERMIND baseline (Farina et al., 2020; Perach et al., 2022), with the exception that MMSE (Molloy & Standish, 1997) was replaced with T-MoCA to assess cognition as the latter has been validated for use over the telephone (Katz et al., 2021). A ‘measures pack’ listing all the answer options (or scales) that would be referred to during the session was sent to participants in advance. Where possible, researchers interviewed the same people they had interviewed at baseline.

### Methods for the nested qualitative study of researcher experience

In October 2020, each of the nine researchers involved in quantitative data collection for DETERMIND-C19 were invited by email to take part in a qualitative telephone interview about their experiences. An information sheet set out the purpose of the interviews and stressed that participation was voluntary and all data would be treated anonymously. Those who consented were interviewed by one of two qualitative researchers (KG and JD) who worked on the wider DETERMIND study but were not directly involved in the quantitative data collection. A third researcher (BH), who had a role in managing the researchers, was involved in study design, recruitment and analysis but did not conduct any interviews. This nested study had HRA and ethical approval as part of the wider DETERMIND study (REC 19/LO/0528; IRAS 261263).

The qualitative interviews with researchers took the form of an extended conversation; following the ‘responsive interviewing’ approach (Rubin & Rubin, 2012). The topic guide ensured we covered, as a minimum: recruitment; experiences of collecting quantitative data over the telephone compared to face-to-face; the use of standardised measures over the telephone; the impact of mode on relationships/rapport; support provided to the researcher; and any other reflections. The interviews started with a general opening question such as ‘*What’s it been like conducting the interviews with people with dementia and their carers over the phone/remotely rather than face to face?*’ and moved on to more focused questions and prompts such as ‘*Have you been able to create a rapport with the participants? What have you done to facilitate this?*’ and ‘*How does this compare with trying to create rapport when face-to-face?*’ Participants’ answers were probed to ensure a thorough understanding of each issue raised. We had originally planned to explore researchers’ experiences of collecting data by telephone *and* video-call, but only a small number of participants with dementia chose video-call, so our focus settled upon the experience of telephone data collection.

Interviews with researchers were audio-recorded and fully transcribed by professional transcribers. Data were managed using the Framework approach (Ritchie & Lewis, 2003) and analysed using reflexive thematic analysis. We took an ‘experiential’ approach, focussing on the meanings and experiences articulated by participants, underpinned by a hermeneutics of empathy (Braun & Clarke, 2021). KG and JD charted the transcripts into an Excel framework with a row per participant and columns with topic headings derived from initial familiarisation. Together with BH, the content of columns was then considered and refined into reflexive themes (‘patterns of shared meaning’ - Braun & Clarke, 2019) conveying the expressed experiences of participants.

## Results

Seven of the nine invited researchers consented to take part in a qualitative interview (labelled A-G). The remaining two researchers did not respond to the invitation.

A table of demographic information and years of experience working in healthcare and/or research for the seven included researchers is shown below (Table 1).

**Table 1.** Researcher demographics and years of relevant experience.

Age band	Gender	Ethnicity	Years in healthcare/research
18–24	Female	White British	1–5 years
18–24	Female	White British	1–5 years
25–34	Male	White British	1–5 years
25–34	Female	British Bangladeshi	1–5 years
25–34	Female	White British	1–5 years
45–54	Female	White British	15+
45–54	Female	White British	15+

All had experience of collecting quantitative data from people with dementia face-to-face and over the telephone as part of DETERMIND and DETERMIND-C19. As such they could make direct comparisons between their experiences of face-to-face and telephone data collection. KG conducted four of the qualitative interviews and JD the remaining three. All lasted approximately 1 hour.

Key demographic information about the people with dementia interviewed remotely is shown in Table 2. Table 2 also shows MMSE scores for these people with dementia (collected face-to-face at baseline) and T-MoCA scores (collected over the telephone during the C-19 lockdown from the same participants) to give an idea of the cognitive abilities of the people the researchers were collecting data from. Type of dementia is also reported where this is known.

**Table 2.** Demographics for people with dementia interviewed remotely for DETERMIND-C19.

	Number	Percent or range	Mean	Response rate (*N* = 93)
Age^a^		Range 58–101 years	80 years	91/93
Gender^a^				91/93
Female	53	57.0%		
Male	38	40.9%		
Missing	2	2.2%		
Ethnicity^a^				91/93
White British	80	86.0%		
Asian	5	5.4%		
Other White	4	4.3%		
Caribbean	1	1.1%		
Other ethnicity	1	1.1%		
Missing	2	2.2%		
Education^a^				87/93
No qualification	24	25.8%		
O/A level	25	26.9%		
NVQ 1-4	17	18.3%		
Degree	14	15.1%		
Post-graduate	6	6.5%		
Other	1	1.1%		
Missing	6	6.5%		
Marital status^a^				91/93
Married/co-habiting	51	54.8%		
Widowed	27	29.0%		
Divorced/separated	9	9.7%		
Single	4	4.3%		
Missing	2	2.2%		
Type of dementia^a^				85/93
Alzheimer’s	53	57.0%		
Vascular	8	8.6%		
Lewy body	4	4.3%		
Mixed	12	12.9%		
Other	8	8.6%		
Missing	8	8.6%		
MMSE at baseline^b^			25 (out of a possible 30)	81/93
21–30	73	78.5%		
10–20	7	7.5%		
<10	1	1.1%		
Missing	12	12.9%		
T-MoCA^c^			13^d^ (out of a possible 22)	61/93
18+	3	3.2%		
10–17	49	52.7%		
<10	9	9.7%		
Missing	32	34.4%	13^d^ (out of a possible 22)	

^a^Collected face-to-face at baseline from the person with dementia or a family carer/informant.

^b^Collected face-to-face from the person with dementia.

^c^Collected over the telephone from the person with dementia.

^d^Equivalent to 24 on the MMSE (Melikyan et al., 2021).

Generally, the researchers considered data collection for DETERMIND-C19 to have gone well, in that most measures were completed with most items answered. However, data collection took around 2 hours and researchers observed that this is a long time to be on a telephone call, especially as it was hard to take meaningful breaks. They noted that visiting in person offered more hooks for building rapport (family photographs, refreshments) and memory triggers (faces, clothing styles) that were not available over the telephone. Not being physically present also meant researchers could not give practical assistance, like making drinks or helping to locate paperwork.

Whilst researchers reported that administering the measures over the telephone *‘tended to go okay’* (Researcher-E), it was not without its challenges. Two key themes were developed to draw together the key challenges raised by the researchers. One relates to the practical requirements of conveying question meaning and scales to people with dementia over the telephone. The other relates to the difficulties researchers experienced in recognising subtle cues over the telephone signalling rising negative emotion in time to prevent distress. We address these themes in turn, before setting out strategies used by researchers to support participants.

### Theme 1 - The telephone adds an extra layer of confusion

Participants reported that the main challenges they experienced in their telephone interviews with people with dementia related to problems with comprehension or retention of the questions and answer scales:

So, those were the worst ones [hardest interviews] where people didn’t understand what I was asking them, couldn’t hold the scales in their head, or weren’t able to use the measures pack to navigate the scales... (Researcher-B)

Such issues, which may also have been present face-to-face, were amplified by the remote mode of administration. Without being physically present to see facial expressions or other body language, researchers noted that the telephone added additional complexity to an encounter that already went against usual conversational norms (with questioners unable to offer explanation and participants required to respond using set answer options), as this researcher summarised: *‘I think it was purely the phone just created that extra layer of confusion.’* (Researcher-E)

The interaction could be further complicated by fatigue or hearing impairment, making it hard to tell in some instances whether difficulties answering questions were due to comprehension or other factors. Some of the measures had multipart structures and required people to hold more than one piece of information ‘in their heads’. The written English in some was complex, but this same language would have been used face-to-face. However, conveying the meaning of complex questions and communicating what was expected of participants was felt to be more difficult over the telephone, in part because participants could not be supported in person with visual aids:

…at baseline [administered face-to-face], because people were finding it confusing and I think it’s more because they couldn’t retain the statements in their head, usually, I just pass over the tablet to them so they can read it, and I’d go at their own pace, whereas over the phone you have no option but to read out the statements... (Researcher-F)

Researchers commented that a lot is expected (cognitively) of participants answering multiple measures using different scales:

The fact you were moving between so many scales ... for some people with dementia, once they got their head round the first scale, it was 1-5, and it was, I don’t know, “Not Applicable” to “Fully Applicable”, the next scale was 1-8 and it was like “Enjoyable” to “Not Enjoyable”... (Researcher-C)

When these kinds of data are collected face-to-face, it is usual to take a pack of response-cards so that researchers can show participants the scale for each measure. Some researchers felt that not having these cards, and more specifically not being able to support participants with dementia to use visual aids in person, significantly impaired their ability to convey meaning in telephone data collection:

…it’s a lot harder to sort of explain the scales over the phone, … you’ve got a question, a scale and then they might need to be reminded of the scale, then they might need to be reminded of the question, so it’s definitely, it’s just harder to visualise for them over the phone (Researcher-G)

In place of the usual response cards, a ‘measures pack’ was sent to each participant in advance of the telephone interview conducted for DETERMIND-C19. This was a 10 page document listing each of the scales relating to each of the measures to be used. Researchers felt that these had generally worked well for carers in the study, but participants with dementia sometimes found them difficult to navigate: they could become ‘lost’, not knowing where to go next or moving on when they were not required to. As a result, rather than acting as an aid to manageability, the packs could for some participants add an additional layer of confusion to the interaction:

…they didn’t know where they were, they didn’t know which scale they were meant to be on, and they found it difficult moving through the sequence of scales, because they weren’t in different colours, you couldn’t say “the red one, the green one”… (Researcher-C)

… they’d sometimes read one and then skip down to the next scale, and you’d have to remind them that we’re on the one above ... we did change things around at one point and then suddenly we were jumping from one page to the other, and that just added a whole other level of confusion… (Researcher-D)

Some of the researchers reported abandoning the measures packs altogether for some participants, resorting instead to reading out the scales repeatedly:

…I was saying “Go to page 8”, whatever, “It’s there” and then people would say “No, it’s not on my page 8” and then …it got abandoned a bit and I ended up just saying “I’ll read out the options” and sometimes people said “I’ll write them down”, if it was a particularly long measure and then they would just do it that way. (Researcher-E)

Longer scales (with more answer options) seemed harder for participants with dementia to respond to. Where numbers represented answers (e.g. 5 indicates ‘extremely’) this could also be problematic, and researchers reported having to regularly repeat what the numbers referred to. Not only could it be challenging to remember all the points in the scale, but the more points there were, the harder it was for some people to remember the original question:

Having to hold that initial bit in your head whilst going through them, that’s quite difficult, and I think it takes repeating that quite a lot to do it, but again if you’re [doing] that over the phone, and you’ve got 20 or 30 questions for you to go through that same preamble every time, it does take a lot more time. (Researcher-B)

### Theme 2 - Telephone administration can lead to missed emotional cues

The topics raised by several of the measures were observed by researchers to trigger negative emotions in participants:

…if you say “Over the last week, have you felt sad? Have you felt lonely? Have you felt isolated?” they can just bring up a lot of emotions, especially for people at the moment, so especially the people with dementia who live on their own, who haven’t been able to see their family …and then you’re like reminding them… (Researcher-G)

Whilst eliciting emotions in itself was not necessarily considered to be negative (indeed some participants appeared to value the opportunity to talk about emotions), researchers felt it was harder to comfort people over the telephone as they could not make physical contact or see from facial expressions and body language how participants were feeling. Moreover, in addition to reminding people or drawing attention to emotions they already felt, some of the measures (and in some cases the act of questioning itself) seemed to cause frustration or distress anew. In particular, the researchers felt that the measure of cognitive impairment was viewed by participants as a ‘test’ and this could provoke anxiety:

I think a lot of the other questionnaires are about your attitudes and opinions and are far more easily answered. The [measure] is an actual cognitive test, so you’re very much placing people on the spot, and it feels very much like a test… (Researcher-C)

Some researchers felt uncomfortable starting a telephone interview with a cognitive test, sensing this set the wrong tone for the remainder of the interview. For DETERMIND-C19 we used a cognitive test designed to be used over the telephone. Nevertheless, it seemed that communication issues linked to mode may have heightened the discomfort experienced by some participants, whilst at the same time making it harder for researchers to gauge if and when they were experiencing discomfort:

…they can become quite distressed because you ask them a memory test over the phone, which sometimes they can’t hear properly, but you can’t tell that they can’t hear properly unless they say something, and you’re giving them all these instructions, which although you read it out exactly how they’re sort of supposed to be read out, sometimes they might not understand and it can be quite frustrating and a bit distressing for them. So, that one often causes quite a lot of distress. (Researcher-G)

Being physically apart from the participant and so not being able to see if or when they were struggling made it harder for researchers to respond to participants’ needs:

…if you’re sitting in a room with someone, or looking at someone you can see that they’re thinking, rather than struggling. …So, just knowing how long to give somebody, before you jump in and move on, or before you jump in, ruin their concentration and spoil their kind of their ability to respond. That’s more difficult. (Researcher-C)

I had a few instances where people broke down, sort of without warning really. I guess for me as a practitioner, face-to-face, I possibly could have picked up on cues, you know, maybe leading up to someone becoming very emotional, whereas over the phone it’s sort of, it just happened, without sort of any sort of prior warning... (Researcher-E)

This risk of missing the cues that signalled participants’ distress was a common concern running through the researcher interviews. In particular, they were concerned that they would pursue a line of questioning on the telephone when, had they been able to see and gauge better the reaction of the participant, they would have stopped or changed tack:

…I could hear in her voice that she was getting upset, so I said “Do you want to stop?” And the carer said “Yes, she’s actually very upset, now” and she started crying and she was very upset, and I just felt really awful, ...I just think that if I had been there, and I had seen her body language, and the way she was reacting, I probably wouldn’t have gone on as long, she probably wouldn’t have got stressed. (Researcher-A)

**…**like their body language might have slightly changed when you said it, you might think “Mmm, maybe I won’t ask it this second time,” but because you can’t see it, you might ask it a second time and that might be when it really sort of triggers and that’s when they really become upset. (Researcher-G)

Most researchers expressed a clear preference for face-to-face data collection with people with dementia, feeling that this gave a better experience for both participants and researchers. However, we have seen in recent times that face-to-face data collection is not always an option. In the final section we turn to the strategies used by the DETERMIND researchers to overcome challenges and make telephone interviewing a good enough mode of data collection.

### Researcher strategies to convey meaning and prevent distress

A number of practical strategies were employed by researchers to support people with dementia faced with the ‘extra layer of confusion’ presented by telephone data collection. Without the option of showing participants questions to give them a better idea of what was expected, some researchers resorted to dividing questions in two so that participants could work through each stage in turn:

So, what I do is I split it up into two, so I say “If you’ve experienced these symptoms, if you tell me ‘yes’ and then if you tell me ‘yes’ I’ll ask you the follow up, which is whether you’ve been distressed by them. (Researcher-B)

Sometimes a researcher would simply move on if a person was struggling with a question or measure, meaning the study would have missing data. Alternatively they might ignore the scale and ask participants to answer verbatim. In this case the researcher would have to decide for themselves where on the scale to place the answer, adding a level of interpretation not envisaged in most measure designs. Other times a combined approach was taken, whereby researchers initially took a verbatim answer, and then offered a truncated scale to reduce the number of options the person picked from:

It was easier to say, read out the question and for them to give an idea of whether they agreed or disagreed, and then to kind of read out that end of the scale, if that makes sense…So, you’re kind of making a judgement, but you’re giving them fewer options… (Researcher-C)

…you end up splitting the scales a little bit. Although you try and do the scales the normal way first, if they really aren’t able to hold that information for too long, it ends up being easier just to do it like that. (Researcher-G)

In theory, sending a measures pack out in advance could overcome some of the problems with scales, but as highlighted above, many participants found these packs difficult to navigate. A number of changes to the packs were suggested by researchers to make them more user-friendly in future, including clearer labelling, large print, better spacing and colour coding. Ensuring such aids are accessible would be particularly important since those most likely to benefit from them are also those most likely to have problems navigating them:

It was definitely more difficult for the person with dementia [to navigate the measures pack], but it’s also more difficult for the person with dementia to remember a scale if they don’t have it in front of them…. So, I think it’s extra useful for a person with dementia but it just could be presented in a much clearer way... (Researcher-A)

After experiencing participants becoming unexpectedly distressed, the researchers began checking-in more to ensure their lines of questioning were not leading to distress: ‘*I was quite careful then from then, to kind of say like, “How is this? Is this okay?*”’ (Researcher-A). Most found that with greater awareness of the potential for distress, and regular checking-in, participants’ wellbeing was supported. However, this required some skill and a delicate *‘balance of not wanting to irritate people by constantly asking them if they’re all right’* (Researcher-D). This could be further complicated by participants’ desire to present well and cover any distress they may be experiencing:

… they might want to just say, “Oh, yeah, yeah, I’m fine,” but they’re not actually okay, whereas at least if you’re face-to-face you can see, like, they’re body language would change. (Researcher-G)

Researchers stressed the importance of sensitivity towards the needs of participants. It was felt, for example, that the impact of the cognitive test could be mitigated by introducing the measure sensitively rather than launching in, but this meant deviating from the official script. Prior knowledge of the participant (from patient notes or having met in person before) was felt to be particularly helpful, and researchers explained that they might draw on what they knew about participants to comfort them if they did become upset.

## Discussion

It is rare that large quantitative studies like DETERMIND open a window onto their data collection processes and encourage the kind of deep reflection entered into by the researchers interviewed for this study. In a review of 88 studies of aphasia treatment, Richardson et al. (2016) found that whilst 57% reported some elements of the assessment process - such as which measures were used or the qualifications of assessors - not one of the reviewed studies included information about what happened when data were collected. The DETERMIND programme was designed to incorporate nested studies exploring researcher and participant experiences. When the COVID-19 pandemic hit and planned fieldwork schedules were disrupted, the DETERMIND team took the opportunity to collect data from their established cohort to assess the impact of the pandemic and associated lockdowns. We also took the opportunity to explore new modes of data collection adopted through necessity.

Despite data collection going well in general, two key challenges were identified: (1) the telephone introduced an extra layer of confusion to an interaction that could already be cognitively challenging; and (2) the demands on participants, together with the content of the measures themselves, could be upsetting, but researchers collecting data over the telephone found it difficult to pick up on cues indicating rising emotion in a timely manner. This echoes previous studies which have noted that standardised measures can raise upsetting issues (Clarkson et al., 2021; De Vries et al., 2014; Gridley et al., 2016) and that cognitive assessments in particular can be experienced by people with dementia as ‘humiliating’ (Hellström et al., 2007, pp. 615–617). One risk in telephone interviewing is that non-verbal cues indicating distress are less detectable by researchers and may be missed until it is too late to avert negative impact. A potential solution could be further training for researchers undertaking telephone data collection to help them detect emotional cues earlier. For this to be useful, however, agreement would have to be reached on how best to respond.

We outlined the strategies researchers developed to convey meaning and prevent distress when collecting data over the telephone for DETERMIND-C19. Guides to qualitative research with people with dementia have repeatedly emphasized the importance of flexibility, creativity and responding to the needs of the person with dementia (Cascio & Racine, 2018; Keady et al., 2017; Novek & Wilkinson, 2019; Phillipson & Hammond, 2018). In contrast, standardised interviewing guidance stipulates that researchers stick strictly to scripts and provide only non-directive prompts in order to avoid interviewer bias (Fowler & Mangione, 2011). The researchers we interviewed were aware of this guidance, but nevertheless described working flexibly and employing person-orientated strategies to support the participants they were interviewing (Silva and Cascio, 2020). At face value their approach seems to go against the requirements of quantitative data collection. On closer inspection these adaptations may be a sensible and sensitive approach to navigating the mis-fit (Webb et al., 2020) between the conventions of standardised data collection and the needs of people with dementia. Even face-to-face, standardised data collection involves an encounter that can be experienced as interactionally strange (Suchman & Jordan, 1990) and, as Phillipson et al. note:

‘Conventional research approaches, such as questionnaires, surveys, and interviews, rely on advanced language and communication skills, recall, abstraction, and verbal reporting all of which are particularly difficult for older people with dementia and cognitive impairment to engage in, comprehend, and manage’ (2019, p. 9)

Over the telephone, these difficulties are accentuated and the researchers in our study therefore made trade-offs. Such trade-offs may not only serve an ethical objective, they could be the very thing that makes data collection from certain groups possible (Hasek, 2015; Isaksson et al., 2007; Jones et al., 2020; Krohne et al., 2013). De Vries et al. (2014) noted that standardised measures frequently raised sensitive topics but allowed little opportunity for those administering them to explore or acknowledge emotional cues from participants. Answers to emotive questions like ‘*I sometimes feel that life isn’t worth living*’ were simply recorded without reaction, and as the interviews proceeded, some participants became increasingly despondent and less communicative. De Vries et al. concluded that strict adherence to standardised delivery could ‘*deny and disacknowledge emotional expression*’ (p. 137) in older people and suggested that this could be both detrimental to the wellbeing of participants and might impair the validity of the data collected, as participants became less engaged. They recommend a more supportive approach that focusses on communicating the meanings of the questions but still allows for systematic collection of data, drawing on Suchman and Jordan (1990) who argue that strict standardisation in the survey interview risks supressing the ‘interactional resources’ that, in ordinary conversation, help us to interpret and convey meaning.

There is some evidence from survey methodologists that a more supportive approach may not lead to the reductions in data quality hitherto predicted. Conrad and Schober have repeatedly demonstrated that a conversational approach, which allows researchers to clarify question meaning when they suspect respondents have misunderstood, leads to improved question interpretation and response accuracy over standardised interviewing and does not lead to interviewer bias (Conrad & Schober, 2000; Schober & Conrad, 1997; West et al., 2018). This has been demonstrated on the telephone as well as face-to-face, and with different populations, including those who may require more support to interpret interviewer instructions. There have, however, been no studies directly examining the effectiveness of conversational interviewing with people with dementia (Conrad & Schober, 2021) and this is therefore a gap to be addressed.

Guides to good practice in working with other participant groups have tackled the question of whether and how much support to provide respondents with additional needs. Guidance for practitioners working with adults with learning disabilities on the Increasing Access to Psychological Therapies programme (IAPT - delivered by the English NHS) specifically advises practitioners collecting outcomes data to break questions with multiple components into smaller chunks and deliver them one at a time (Dagnan et al., 2015). This is presented as a ‘reasonable adjustment’ required to support inclusion. Whilst the authors note that question wording should only be changed if absolutely necessary, and then as little as possible, they also note that failure to support those who have difficulties completing the assessment is ‘highly likely’ to lead to withdrawal from the programme. Reasonable adjustments to data collection protocols in dementia research might therefore be justified both as a means to promote equality and as a strategy to minimise withdrawals.

Phillipson et al. (2019) argue that the specific needs of people with dementia require the adaptation of traditional research methods to support inclusion. They prompt us to ask whether methods are manageable, meaningful, and comprehensible and reason that, without this attention to the needs of participants, we risk impairing wellbeing *and* collecting poor quality data. The DETERMIND researchers drew upon interactional resources to convey meaning and prevent distress – striking a balance between the needs of participants and the requirement for reliable data. In this sense they took a person-orientated approach characterised by a focus on researcher-participant relationships, respect for personhood and individualization, whilst endeavouring not to compromise data integrity (Cascio & Racine, 2018). It is likely that such a balance is actively negotiated in many studies (Houtkoop-Steenstra & Houtkoop-Steenstra, 2000) but without exploring and discussing the data collection process more openly we cannot begin to understand the need for, or consequences of, such compromises, either for data quality or for the well-being of the people involved.

### Strengths and limitations

This study gives a rare insight into the experiences of individual researchers administering standardised measures over the telephone to people with dementia. Whilst previous attention has been given to the psychometric properties of measures (Stoner et al., 2018) and risks of bias (Fowler & Mangione, 2011), very little is known about the ‘lived experience’ of measure administration (Abendstern et al., 2019) or the strategies used by researchers to combat interactional strangeness (Suchman & Jordan, 1990). Nevertheless, our research was opportunistic and looked only at data collection from the perspectives of researchers. Interviews with people with dementia themselves, and observations of the processes involved, would help to more fully understand this phenomenon and its impact on both participant wellbeing and data quality.

It should also be remembered that our data were collected during the coronavirus pandemic, a very unusual time, and one where people with dementia may have been particularly vulnerable due to services and supports not being available (Wheatley et al., 2022). It is hard to be sure, in this context, whether participants were more susceptible to negative emotions than they would be in usual times. However, this is not the first study to flag the potential emotional burden of standardised measures for older people and people with dementia (De Vries et al., 2014; Hellström et al., 2007). What this paper adds is the insight that telephone interviewing may make minimising and managing this more difficult, whatever the context.

Finally, we were only able to examine the use of telephone data collection, since very few people opted to use video. It will be important to learn in future studies whether video technology can overcome the problem of missed emotional cues raised in this study or if it is necessary to be physically present in order to pick-up and respond to these in a timely way.

## Conclusions

We gathered insights from recent experiences of researchers who found themselves collecting standardised data over the telephone from people with dementia, where previously these data had been collected face-to-face. Whilst particularly relevant in dementia research, the findings may be of value to those planning any research where telephone quantitative data collection is being considered or is the only viable option. The researchers we interviewed felt that the telephone added an extra layer of confusion to an encounter that was already challenging for some. They also observed that questioning could trigger frustration and even distress in some participants, but felt less able to detect or respond to this than when physically present. The strategies researchers used to convey meaning and support participants may not conform to the strict conventions of standardised measurement, but they were informed by person-oriented principles and the desire to ensure that research participants were ‘emotionally safe’ (Flemington et al., 2014). It is likely that other large scale dementia studies will, or already have, faced similar challenges. These must be discussed openly and honestly for the field to develop and to ensure the meaningful, ethical involvement of people with dementia in future research.
